# Parallel Increase in Childhood Anorexia Nervosa and Early Puberty During the COVID‐19 Pandemic

**DOI:** 10.1002/eat.24556

**Published:** 2025-09-27

**Authors:** Beate Herpertz‐Dahlmann, Astrid Dempfle, Stefan Eckardt, Josef Neulen, Kelly L. Klump

**Affiliations:** ^1^ Department of Child and Adolescent Psychiatry, Psychosomatics and Psychotherapy RWTH University Aachen Germany; ^2^ Institute of Medical Informatics and Statistics Kiel University Kiel Germany; ^3^ Techniker‐Krankenkasse (Techniker Health Care Service), State Representation North‐Rhine‐Westphalia Düsseldorf Germany; ^4^ Department of Geriatric Medicine RWTH University Aachen Germany; ^5^ Department of Psychology Michigan State University East Lansing Michigan USA

**Keywords:** anorexia nervosa, anxiety disorders, atypical anorexia nervosa early onset, childhood precocious puberty, COVID‐19, depressive disorders, early puberty, environmental stressor, pandemic

## Abstract

**Objective:**

During the COVID‐19 pandemic, an increase in anorexia nervosa (AN), specifically childhood AN, as well as in central precocious puberty (CPP) and early‐onset puberty (EOP), was reported. The aim of this study was to explore whether there was a population‐level association between increases in both disorders and to discuss possible underlying causes.

**Method:**

Data were retrieved from the largest health insurance institution in Germany comprising approximately 3.5 million children between 0 and 14 years for the years 2019–2023. All female cases with a diagnosis of AN/atypical AN and those with CPP/EOP according to ICD‐10 were included. To investigate possible specificity of associations, we also examined associations with depressive disorders (DD) and anxiety disorders (AD).

**Results:**

Decreasing and increasing numbers of cases with EOP, CPP, and childhood AN/atypical AN showed a similar pattern during the COVID‐19 pandemic. The number of diagnosed cases of AN/atypical AN combined with either CPP (Spearman's *ρ* = 0.45; *p* = 0.02), EOP (Spearman's *ρ* = 0.60; *p* = 0.003), or combined CPP/EOP (Spearman's *ρ* = 0.53; *p* = 0.008) in this time span was highly and significantly correlated. Associations with CPP/EOP were generally stronger for AN/atypical AN than for DD (Spearman's *ρ* = 0.45; *p* = 0.02) or AD (Spearman's *ρ* = 0.29; *p* = 0.11).

**Discussion:**

The highly increasing prevalence of childhood AN, EOP, and CCP may reflect pandemic‐associated stress and lifestyle changes and/or their effects on reproductive functioning. Pre‐ and peripubertal girls seem to be especially vulnerable to these environmental stressors and might react with important physical and mental impairments.

**Summary**

- Prevalence rates of CPP/EOP and early onset AN/atypical AN during the pandemic followed a parallel course with highly increasing rates of both disorder groups.
- Increases in prevalence rates of both disorders were also associated with increases in depressive and to a lesser extent with anxiety disorders, but associations between CPP/EOP and AN/atypical AN tended to be stronger.
- Findings suggest that COVID‐19 induced stress, changes in lifestyle, and/or effects on reproductive functions may have impacted both CPP/EOP and AN/atypical AN.
- Young girls seem to be especially vulnerable to environmental stress and strains. Prevention and support programs for EDs should be extended to the childhood group of 10–14 year olds.

## 1 Introduction

Shortly after the beginning of the COVID‐19 pandemic, many studies from all over the world demonstrated an increase in referrals of patients with eating disorders (EDs). Several investigations have also reported an important shift to childhood anorexia nervosa (AN), in particular in the age group between 10 and 14 years (Auger et al. 2023; Toigo et al. 2024; Hansen et al. 2024).

In our own investigations of a large, nationwide and representative sample in Germany, the admission rate ratio (i.e., the difference between the admission rates in the pre‐COVID‐19 era in 2019 and the peak of the COVID‐19 period in the beginning of 2022) was slightly greater in 9–14‐year‐old children than in adolescents (ratio = 1.40 vs. 1.32). In 2023, the admission risk declined for adolescents/young adults but remained high for children ≤ 14 years. According to our results, the risk for children to be admitted to the hospital because of AN or atypical AN increased by 42% in the first half of 2023 compared to 2019 (Herpertz‐Dahlmann et al. 2022, 2024).

During the COVID‐19 pandemic, several endocrinological treatment centers also reported an important increase in admissions for central precocious puberty (CPP) and early‐onset puberty (EOP). Puberty in girls is deemed physiological when breast development starts between 8 and 12 years of age (Abreu and Kaiser 2016). Based on a mean pooled analysis from different countries, the current mean age of menarche is approximately 12.5 years (Maione et al. 2021; Sørensen et al. 2012). CPP is defined as an onset of breast development in girls (i.e., breast bud palpable under the areola corresponding to Tanner breast stage “B2,” otherwise known as thelarche) before 7.5–8 years of age (Zevin and Eugster 2023), while EOP is diagnosed with Tanner stage “B2” between the eighth and ninth years of life (Baehr et al. 2023). In boys, testicular enlargement before 9 years is defined as CPP, while enlargement between 9 and 10 years is defined as EOP. The prevalence of CPP in girls is up to 15 times greater than that in boys (Zevin and Eugster 2023).

The majority of reports showing increases in CPP and EOP came from Italy, a country that was very early hit by the pandemic (Indolfi and Spaccarotella 2020). In a tertiary academic center in Genova, Italy, the number of cases per month diagnosed with rapidly progressive CPP more than doubled (1.44 vs. 3.8) between March 2020 and the end of June 2021 compared with the number of cases per month diagnosed between January 2016 and March 2020 (Fava et al. 2023). In an endocrinological unit in Rome, a significant increase in CPP was observed in 2020, as well as in 2021 but with a lesser increase (Chioma et al. 2023). Other reports came from Turkey (Acar and Özkan 2022), Japan (Matsubara et al. 2023), and the United States (Trujillo et al. 2022) (for a review, see Alonso 2024). In Germany, the number of girls diagnosed with CPP or EOP in 18 tertiary centers of pediatric endocrinology increased from 607 in 2019 to 1006 in 2021, an increase of approximately 66% (Baehr et al. 2023). Interestingly, the age at presentation for CPP was lower during the COVID‐19 associated restrictions than before the pandemic (Nguyen et al. 2024).

Several studies by Klump (2022) suggested that the activation of ovarian hormones during puberty might contribute to a heightened risk for ED, potentially via hormone‐mediated changes in gene transcription. Nonetheless, a clear connection has not yet been demonstrated, partially due to the difficulty in examining puberty/AN links given the impact of starvation on the onset and progression of pubertal development. Consequently, the aim of this study was to indirectly investigate potential puberty/AN links by examining whether there was a correlation between the increasing prevalence of childhood AN/atypical AN, CPP, and EOP during the COVID‐19 crisis using health insurance data on over 3.4 million child patients in Germany. In addition, we wanted to examine whether increasing rates of adolescent depressive disorder (DD) and anxiety disorder (AD) ran in parallel to the co‐occurrence of CPP/EOP and AN to determine a potential specificity of associations. Because the health insurance data in this study are anonymous, we were unable to link cases across diagnostic groups to examine trajectories of CPP/EOP and AN within individuals. Thus, our findings speak to whether the overall population prevalence of these conditions exhibits similar trajectories across the pandemic rather than the co‐occurrence and trajectories of the disorders within individuals (e.g., CPP preceding AN).

## 2 Methods

### 2.1 Study Design

Data collection and evaluation have been described in detail previously (Herpertz‐Dahlmann et al. 2022, 2024). Patient data were retrieved anonymously from the largest statutory health insurance institution in Germany, with 28.5 million members and a market share of 38.4% (https://www.vdek.com/presse/daten/_jcr_content/par/publicationelement1479644990/file.res/vdek_basisdaten_2024.pdf, assessed January 18, 2025), and delivered to the first and second author. This dataset comprised between 3.4 million and 3.5 million children between the ages of 0 and 14 years for the years 2019–2023. Although sociodemographic factors at the individual level are not available in this dataset, the socioeconomic status of the sample was similar to that of the general population in Germany, with a somewhat greater percentage of higher‐income classes in our sample compared to the population of Germany (10% vs. 5%; Ministry of Health, Germany; Zahlen und Fakten zur Krankenversicherung [BMG]). In Germany, every individual is committed to being a member of health insurance.

### 2.2 Participants

**FIGURE 1:**
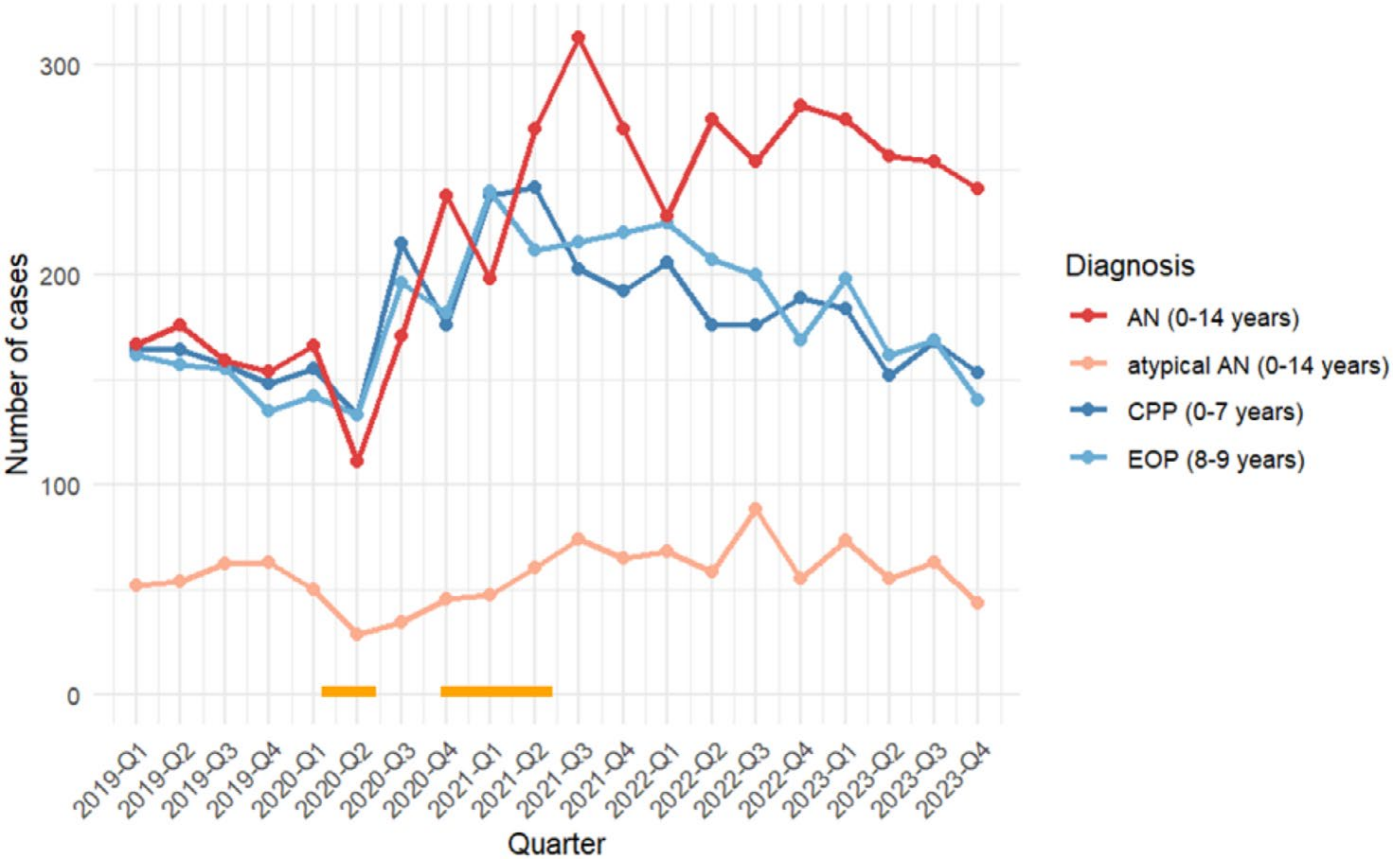
Course and number of cases with childhood AN and atypical AN (0–14 years), precocious puberty CPP (0–7 years), early‐onset puberty EOP (8–9 years) or CPP/EOP combined (0–9 years) per quarter of the years 2019–2023. Percentage of atypical AN across the years of the investigation: 26.04% overall prevalence (atypical AN *n* = 231 vs. AN *n* = 656) in 2019, 18.62% (atypical AN *n* = 157 vs. AN *n* = 686) in 2020, 18.97% in 2021 (atypical AN *n* = 247 vs. AN *n* = 1051), 20.6% in 2022 (atypical AN *n* = 269 vs. AN *n* = 1037), and 18.57% in 2023 (atypical AN *n* = 234 vs. AN *n* = 1026). Horizontal orange lines denote times of school closures in Germany (3/2020 to 5/2020 and 11/2020 to 5/2021).

As the number of males in precocious puberty and EOP was negligible in our sample, we recorded only female cases. A case was defined as a single hospital record. For childhood AN, all female cases ≤ 14 years who were hospitalized with a diagnosis of AN (ICD‐10 *F* 50.0) or atypical AN (ICD 10 *F* 50.1) and who were members of the health insurance institution VdEK were included. Median age and age quartiles for childhood AN/atypical AN were constant across the investigation period (AN and atypical AN, respectively: mean age: 13 (first and third quartiles: 13; 14), AN and atypical AN combined: mean age 13 (13; 14)). In this sample, AN was more prevalent than atypical AN in all study years (Figure 1).

For CPP and EOP (ICD‐10 E 30.1), we created two nonoverlapping groups: all female cases diagnosed with CPP until and at the age of 7 (to be most conservative) and those diagnosed with EOP at the age of 8–9. A combined group of CPP and EOP (i.e., age 0–9) was also considered in the analysis (median age and first/third age quartiles CPP: 5 (5; 7); EOP 8 (8; 9) combined: 8 (6; 8)). In addition, we assessed all female cases with DD (ICD‐10, *F* 32 and *F* 33) and AD (ICD‐10, *F* 40 and *F* 41) that were ≤ 14 years (median age and first/third age quartiles DD: 14 (13; 14); AD: 13 (12; 14)).

### 2.3 Early AN Definition

Hospital discharge diagnoses coding AN or atypical AN were obtained from all pediatric and child and adolescent psychiatric hospitals in Germany between January 2019 and December 2023. Focusing on these discharge diagnoses ensured that every diagnosis was made by an experienced medical team, but we did not include outpatients, as these AN diagnoses are often not substantiated. In the data storage system of the health insurance, diagnoses of AN or atypical AN were made according to the ICD‐10 (*F* 50.0, *F* 50.1) and the German guidelines for EDs (Herpertz et al. 2019). In these guidelines, the weight threshold for AN in children and adolescents is defined as a BMI at or below the 10th age‐specific percentile. According to the ICD‐10, atypical AN was diagnosed when one essential ICD‐10 criterion was lacking; in the majority of cases, the weight criterion was not met (according to the definition in the DSM‐5). As the data storage system of health insurance categorizes all patients ≤ 14 years into one age group, we decided to include every discharge diagnosis of AN/atypical AN given at or below 14 years in the childhood‐onset group. This age cut‐off is justified by the meta‐analysis by Solmi et al. (2022), in which the proportion of patients diagnosed with AN by 14 years was only 10.6%, whereas the peak age at diagnosis of AN (male and female) was 15.5 years.

### 2.4 CPP and EOP Definitions

To ensure a reliable diagnostic process, and because CPP and EOP diagnoses are normally made in Germany after a thorough physical assessment in specialized pediatric centers, all diagnoses of CPP and EOP (coded E 30.1 according to ICD‐10) made in outpatient clinics of pediatric hospitals from 2019 to 2023 documented in the data storage system of the health insurance were recorded. A diagnosis of EOP was given with a breast development “B2” between the ages of 8 and 9 (Baehr et al. 2023) (see above). In contrast to AN, for which many adolescents in Germany are admitted for inpatient treatment, this is not the case for CPP or EOP. Patients admitted to inpatient treatment for CPP were so few (e.g., *n* = 162 in 2019 for the whole country of Germany) that we decided not to consider them because they most likely had additional medical problems.

### 2.5 DD and AD

Similar to AN/atypical AN, hospital discharge diagnoses of DD (ICD‐10, *F* 32 and *F* 33, without bipolar disorder) and of AD (ICD‐10, *F* 40 and *F* 41) were obtained for female patients ≤ 14 years from all pediatric and child and adolescent psychiatric hospitals in Germany during our investigation period.

### 2.6 Statistical Analysis

Spearman's rank correlation coefficients (*ρ*) were used to assess associations between the number of female cases of childhood AN/atypical AN and either CPP (0–7 years), EOP (8–9 years), or CPP/EOP combined (0–9 years) *per quarter* during the Covid‐19 pandemic (from Q1/2019 to Q4/2023) as well as associations between AN/atypical AN and CPP/EOP with DD and AD during the same investigation period.

## 3 Results

The decreasing and increasing numbers of cases with CPP, EOP, and early AN/atypical AN demonstrated a similar trajectory from pre‐COVID‐19 (first through the fourth quarter of 2019), throughout the pandemic (first quarter 2020 through the fourth quarter 2022), and post‐COVID‐19 (first to the last quarter of 2023). These trajectories are depicted in Figure 1 for the entire group of female patients, with separate lines for CPP (≤ 7 years old), EOP (8–9 years), AN, and atypical AN.

The number of diagnosed cases was highly correlated for AN and CPP, AN and EOP, and the combined CPP/EOP and AN/atypical AN groups (Table 1). Associations between CPP and EOP and atypical AN were somewhat attenuated (see Table 1), but the associations were significant (*p* = 0.02) or approached significance (e.g., *p* = 0.06) for EOP and the combined CPP/EOP group.

**TABLE 1:** Spearman's *ρ* correlations (*p* values) between CPP, EOP, AN, atypical AN, and depressive and anxiety disorders.

Disorder	AN	Atypical AN	AN and atypical AN (combined)	Depressive disorders	Anxiety disorders
CPP	**0.51 (0.01)**	0.26 (0.13)	**0.45 (0.02)**	0.32 (0.09)	0.15 (0.26)
EOP	**0.59 (0.003)**	**0.45 (0.02)**	**0.60 (0.003)**	**0.56 (0.005)**	**0.41 (0.04)**
CPP and EOP (combined)	**0.56 (0.005)**	0.35 (0.06)	**0.53 (0.008)**	**0.45 (0.02)**	0.29 (0.11)

*Note*: Statistically significant correlations are noted in bolded font.

Abbreviations: AN = anorexia nervosa, atypical AN = atypical anorexia nervosa, CPP = central precocious puberty, EOP = early‐onset puberty.

**FIGURE 2:**
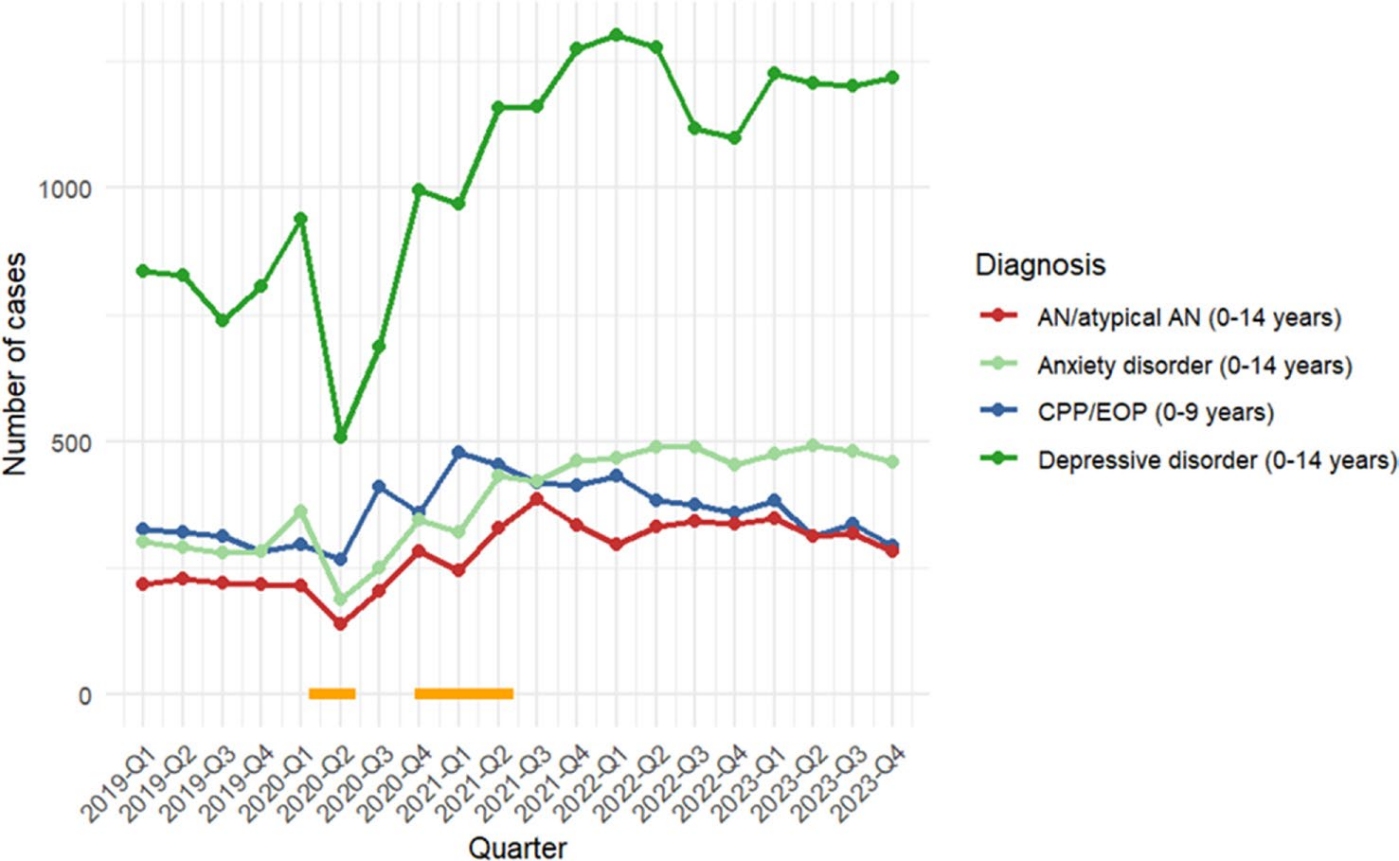
Course and number of cases with childhood AN/atypical AN (0–14 years, combined), CPP/EOP combined (0–9 years), depressive and anxiety disorders per quarter of the years 2019–2023. Horizontal orange lines denote times of school closures in Germany (3/2020 to 5/2020 and 11/2020 to 5/2021).

Looking at the course of the case numbers in both disorder categories, the number of diagnosed cases of early AN/atypical AN increased later than those of CPP and EOP. Although we cannot directly investigate this hypothesis, it may be that it took some time until patients with AN had lost the amount of weight defined by the ICD‐10 criteria. For this reason, we also assessed the correlation between CPP or EOP case numbers in each quarter with AN/atypical AN case numbers in the immediately following quarter (e.g., comparing the case numbers of CPP or EOP in the first quarter of 2020 with the case numbers of childhood AN/atypical AN in the second quarter of 2020 and so on). The correlation between these time‐shifted diagnosed cases of CPP or EOP and combined childhood AN/atypical AN was even greater (CPP Spearman's *ρ r* = 0.70, *p* = 0.0004; EOP Spearman's *ρ r* = 0.75, *p* = 0.0001) and for combined CPP/EOP: Spearman's *ρ r* = 0.75; *p* = 0.0001 (Figure 2).

In terms of DD and AD, there were more consistent associations and trajectories of cases between CPP/EOP and DD than between CPP/EOP and AD (see Table 1 and Figure 2). Nonetheless, correlations between the number of admitted cases for CPP/EOP and DD were somewhat smaller than those observed for CPP/EOP and AN. Perhaps not surprisingly, given high rates of comorbidity, correlations between AN/atypical AN and DD and AD were moderate‐to‐large (Spearman's *ρ* = 0.51–0.75, *p*'s < 0.05).

## 4 Discussion

Several reports from all over the world have pointed to a significant increase in AN (Hansen et al. 2024) and EOP/CPP (Nguyen et al. 2024) in young people during the COVID‐19 crisis. The percentage increase in admission rates was greater in childhood than in adolescent patients with AN (Hansen et al. 2024; Herpertz‐Dahlmann et al. 2024). To our knowledge, we are the first to demonstrate that during the pandemic, both EOP and childhood AN followed a similar chronological sequence. At the beginning of the COVID‐19 crisis in the first quarter of 2020, referrals for the two diagnoses declined; the maximum number of admissions for EOP was reported for the first quarter of 2021, whereas the maximum number of cases diagnosed with childhood AN was reported in the third quarter of 2021. The referrals for both disorders declined in 2022. Both diagnostic groups were assessed in the same population but *independently* from each other. While the number of CPP/EOP cases returned to a pre‐COVID‐19 level, this was not the case for childhood AN, which still presented in our sample with much higher rates in 2023 than in 2019 (Herpertz‐Dahlmann et al. 2024) followed by a small decline in 2024 (unpublished data). This might be due to a general and continuing increase of childhood AN as has been already described by Smith et al. (2023) and van Eeden et al. (2023) before the pandemic crisis. In addition, a greater awareness by clinicians, parents, and teachers might have contributed to a higher detection rate. There is also some evidence in this study that the absolute numbers of admissions for atypical AN in this time frame increased (Herpertz‐Dahlmann et al. 2024). A similar trend was found in Norway (Reas et al. 2025).

The number of CPP and EOP cases increased dramatically in several countries during and after the COVID‐19 associated lockdowns (for a review, see Street et al. 2023). The highest increases in admissions were reported in the third quarter of 2020, and high rates were still reported in 2021, followed by a beginning decline in 2022 (Chioma et al. 2023).

The decline in presentations of both disorders at the beginning of the COVID‐19 pandemic in spring 2020 might be due to carers' precautions not to risk an infection of their child by bringing them into a hospital. Interestingly, in both Germany and Italy, the maximum number of diagnoses in both groups was made during the summit of the COVID‐19 restrictions, with school closures from 3/2020 to 5/2020 and from 11/2020 to 5/2021 (Figures 1 and 2) (Chioma et al. 2023; Herpertz‐Dahlmann et al. 2022).

There was a strong and significant association between the ups and downs in prevalence of CPP/EOP and AN/atypical AN, which was stronger than for the association of CPP/EOP with DD or AD, probably indicating a specific effect. The association between CPP/EOP and AN was more pronounced than that for CPP/EOP and atypical AN, suggesting there are more common causes for AN and CPP/EOP (Watson et al. 2022; Breton and Kaufmann 2025).

Given these significant associations, it is important to consider potential explanations for the co‐occurrence of increasing admission rates for CPP/EOP and childhood AN.

### 4.1 Pandemic‐Associated Stress and Threats

Pandemic‐associated stress and restrictions, which might have been especially important in young age groups (Creswell et al. 2021), could have played a major role in the similar accumulation of both disorders. In systematic reviews including studies from all over the world, an increase in emotional stress during the COVID‐19 pandemic was reported in young people (mean age: 11 years) with significantly increasing depression and anxiety levels (Panchal et al. 2023) as has also been observed in the present study. Age‐specific effects were considerably greater in children and young adolescents (i.e., 11–15‐year‐olds vs. 16–19‐year‐olds), especially during times of stronger restrictions such as school closures (Ludwig‐Walz, Dannheim, et al. 2023).

Importantly, COVID‐19‐associated stress followed by both anxious and depressive symptoms likely contributed to the worsening or emergence of EDs during the pandemic (Zeiler et al. 2021). Patients with an onset of AN during the COVID‐19 pandemic reported significantly greater levels of anxiety and depressive symptoms than did those with an onset of AN before the pandemic (Gilsbach and Herpertz‐Dahlmann 2023; Meneguzzo et al. 2024). While physical stress, such as intense sports training, low‐energy diets, and chronic illness, might delay the onset of puberty (Butenandt 2017), stress and childhood adversities (e.g., adoption, fathers' absence, sexual abuse) have been identified as risk factors for CPP (Nguyen et al. 2024; Zevin and Eugster 2023). According to the hypotheses of several authors, stress‐ or anxiety‐induced neurotransmitter and hormonal level changes could trigger cerebral pathways involved in pubertal development (Barberi et al. 2022; Prosperi and Chiarelli 2023). Italy, as an example, was one of the first countries to report a sudden increase in the number of help‐seeking patients for CPP/EP (Stagi et al. 2020). It had one of the earliest and most severe COVID‐19 infection periods in Europe and was characterized by a very high mortality, with peak mortality rates in spring and autumn of 2020 and in March of 2021, which occurred parallel to the increase in CPP/EOP and AN. During this time, Italy also practiced school closures and strict restrictions on the whole country (https://en.wikipedia.org/wiki/COVID‐19_pandemic_in_Italy; Amante and Balmer 2020; Indolfi and Spaccarotella 2020). Unfortunately, only two studies have assessed pandemic‐associated stress in patients with CPP/EOP. Approximately half of the girls in these two cohorts reported symptoms related to COVID‐19‐related burdens (Baby et al. 2023; Chioma et al. 2022). However, neither study included a control group with normal pubertal timing. The overarching association of AN/atypical AN and CPP/EOP with DD (and to a lesser extent with AD) might support our hypothesis that pandemic‐associated stress contributed to the increasing rates of both disorders. Nevertheless, the link between CPP/EOP and AN was stronger than that between CPP/EOP and depression, which might suggest that there are other factors beyond pandemic‐related stress that contribute to the CPP/EOP and AN/atypical AN associations (please see also below).

### 4.2 Pandemic Effects on Pubertal Development That Then Lead to AN

As has been outlined above, COVID‐19‐associated strain could have induced early pubertal development. In an endocrine unit in the United States, higher levels of ovarian and gonadal hormones, as well as larger ovarian volumes, were found in the group with CPP referred during the pandemic as compared to the pre‐pandemic group (Baby et al. 2023). Puberty is a significant risk period for the development of AN, especially in girls, with a peak age of onset at 15.5 years (Solmi et al. 2022). A prospective investigation to study the influence of gonadal hormones on the development of AN is difficult, as starvation arrests typical pubertal progression, and patients typically delay the beginning of treatment (Watson et al. 2022). Moreover, the low prevalence of AN impedes larger prospective studies. Nevertheless, several studies have assessed the association between the onset of puberty and the development of AN and other EDs (e.g., bulimia nervosa [BN]) and their symptoms (e.g., binge eating, weight/shape concerns, body dissatisfaction). Although the results of puberty/ovarian hormone research for AN have been less consistent than those for BN or binge‐ED (Klump 2013), a more recent genome‐wide association study (GWAS) by Bulik and her group (Watson et al. 2022) revealed a significant genetic correlation between younger age at menarche and early onset AN. The authors postulated that a subgroup of women with EDs might present an “ovarian hormone‐sensitive phenotype”. In a very recent study based on several GWASs of AN and age at menarche, nine shared genomic risk loci were observed between AN and age at menarche, which led the authors to recommend “more puberty‐related variables as a primary research focus” in AN (Breton and Kaufmann 2025).

However, to our knowledge, there are no reports in the literature about CPP as a preceding disorder for AN. Thus, it would be interesting to determine whether there is a greater proportion of patients with early AN and distinctive markers of early pubertal development compared to those with on‐time signs of puberty. Interestingly, the association between CPP/EOP and AN was stronger than that between CPP/EOP and atypical AN. The reason is not quite clear, but possible explanations could be that the clinical phenotype and the definition (especially according to ICD‐10) of atypical AN are more variable than those of AN, which might weaken the association.

Puberty could also have indirect effects via risk factors that are known to predict the development of AN. In our study, there was a significant accordance between the prevalence of CPP/EOP and that of DD, confirming previous studies demonstrating an association between early pubertal timing and internalizing emotional disorders (Barendse et al. 2022). In young people, depression not infrequently precedes AN (Bühren et al. 2014), and our own study results, as well as those from others, demonstrated that the pandemic produced a significant increase in depressive symptoms in this age group (Ravens‐Sieberer et al. 2021). Consequently, it may be that increases in internalizing symptoms during COVID‐19 serve as mediators of associations between CPP/EOP and AN, such that CPP/EOP leads to increased internalizing symptoms that then lead to early AN. By contrast, puberty could act as a moderating factor between the beginning of disordered eating and mood and/or other psychological symptoms, whereby internalizing symptoms are more likely to lead to AN in the presence of CPP/EOP. Supporting this last possibility, Vo et al. (2021) reported stronger associations between mood/personality factors and cognitive symptoms of EDs (i.e., body dissatisfaction, shape/weight concerns) in girls with more advanced pubertal development.

### 4.3 Weight Gain and Changes in Physical Activity During the COVID‐19 Pandemic

Many endocrinologists note that overweight and obesity, as well as a sedentary lifestyle, may contribute to EOP in girls, most likely through the influence of the peptide hormone leptin (Li et al. 2017; Maione et al. 2021; Zevin and Eugster 2023). Significant weight gain and increased obesity rates have been reported in Germany as well as in other regions of the world during the COVID‐19 pandemic, especially in young people (Pfefferbaum et al. 2024; Vogel et al. 2022). Moreover, because of COVID‐19 restrictions, there was a significant decline in children's motion behavior (Ludwig‐Walz, Siemens, et al. 2023).

The increased rates of weight gain could have contributed to the increased admission rates of childhood AN, especially atypical AN. Past studies have reported that young people felt ashamed of assumed or real weight gain during the first months of the COVID‐19 pandemic (Gilsbach and Herpertz‐Dahlmann 2023; Rodgers et al. 2020) and deliberately restricted their food intake and increased their physical activity, with the consequence of weight loss. Although the percentage of admissions for atypical AN versus typical AN was lower during the pandemic, the absolute numbers were higher than before the crisis (see above). The lower percentage could be due to hospitals running out of beds; thus, patients with lower body weights were more likely to be admitted. Interestingly, corresponding to the data found for childhood AN, obesity rates in 12–16 year‐old girls did not decline after the pandemic while these rates did decline in boys and younger children (Berisha et al. 2025).

The increase in obesity rates might only partly explain the co‐occurrence of EOP and childhood AN. While some authors reported an increase in BMI in girls with CPP and EOP during the COVID‐19 pandemic in comparison with the pre‐COVID‐19 years, this finding was not confirmed by others (Chioma et al. 2023; Prosperi and Chiarelli 2023; Umano et al. 2022). In a comprehensive meta‐analysis of 32 studies, no difference in BMI between the pre‐pandemic and pandemic CPP/EOP groups was found (Nguyen et al. 2024). Moreover, a rise in consultations for EOP was already observed during the first months of the COVID‐19 pandemic, when the impact on weight could not have been significant (Stagi et al. 2020).

### 4.4 Nonspecific Association Between AN and CPP

We also have to discuss an association between both disorders due to coincidence, for example, that the high correlation between the observed increases only reflects parallel increments in several and different medical and mental health conditions during the pandemic that are unrelated to each other. Importantly, the attenuated associations between CPP/EOP and AD (and to a lesser extent DD) suggest that there is some specificity in the similar trajectories of CPP/EOP and AN/atypical AN across COVID‐19. Nevertheless, COVID‐19 was associated with increased prevalence of a number of medical and stress‐related conditions. Pandemic‐associated epidemiological findings in medical disorders of childhood and adolescence are contradictory, in part, because data assessed during and after the pandemic have not been frequently published, and if so, only for a few nationalities. Moreover, several interactions between the COVID‐19 infection mechanism and the medical disorder itself were observed. Incidence during the pandemic varied widely between chronic disorders of childhood. For example, decreases in pediatric asthma, atopic skin disorders (Liang et al. 2024), and Crohn's disease (Kasem Ali Sliman et al. 2025) were observed while increases were reported in juvenile rheumatoid arthritis, Type 1 Diabetes, and Graves' disease (D'Souza et al. 2023; Donner et al. 2023; Kasem Ali Sliman et al. 2025; Vlădulescu‐Trandafir et al. 2024). Thus, it is not possible to determine whether the association between CPP and AN is specific or only mirrors parallel increases in different disorders of childhood. Moreover, a direct influence of the virus interacting with neurotransmitter or endocrinological functions cannot be ruled out. In several endocrine disorders, such as Graves' disease, a direct association between acute COVID‐19 infections and organ dysfunction has been demonstrated (Anbardar et al. 2025). Nonetheless, in a review of 22 studies, none of them reported that girls with CPP/EOP had indications pointing to a confirmed or suspected COVID‐19 infection (Alonso 2024).

## 5 Limitations

This study has several limitations. Because of the coding system of German health care institutions, our results are based on the ICD‐10, which is no longer the current classification system. However, this classification system remained stable throughout the entire observation period between 2019 and 2023, so the diagnoses of AN/atypical AN and CPP/EOP during this time span were based on the same criteria. While discharge diagnoses of AN/atypical AN, DD, and AD are very reliable, this may not be the case in outpatient diagnoses of CPP/EOP. However, as these diagnoses were made in pediatric hospitals, and endocrinological centers worldwide reported the same epidemiological development as that reported by us, our data seem to be reliable; nevertheless, our study is not an epidemiological survey but rather an evaluation of the development of clinical populations, especially CPP/EOP and AN/atypical AN, during the COVID‐19 pandemic.

We want to emphasize that both samples (and also those of the other disorders) were assessed independently. As this was a cross‐sectional and anonymous investigation, we could not assess the impact of individual stressors, anxiety, or depressive symptoms to identify their role in the relationship between CPP/EOP and AN. Moreover, we could not follow the course of the disorders in single patients during later development (i.e., we could not determine whether patients with EOP were more likely to develop AN later). This question should be explored in countries where mental and physical disorders are included in national registers, for example, in Scandinavia so that an individual follow‐up is possible.

## 6 Conclusion

Our investigation demonstrated that there was a highly significant association between the course of CPP/EOP and early AN during the COVID‐19 crisis. One explanation could be that the increasing incidence of both disorders in children was potentially associated with pandemic‐induced stress, threats, and restrictions. As both samples were assessed independently, this does not prove causality and may be the result of a pure coincidence. This could also be the consequence of a pandemic‐induced change in lifestyle. Provided—but not proven—that sex hormones play a role in the development of AN, the increase in the prevalence of both disorders might also be the result of earlier puberty provoked by COVID‐19‐induced stressful life events. Thus, our observation could imply an association between an earlier onset of puberty during the past decade and an increasing number of early AN cases. However, as pubertal development is suppressed in AN, this cannot be proven. The aim of this report was to open a new research window to assess the impact of pubertal development on the development of early AN. Further studies are necessary to reveal an association between the onset of puberty and the onset of AN. However, our results point to a high physical and mental vulnerability of the developing female organism, which should result in early detection and treatment of both disorders.

## Author Contributions

**Beate Herpertz‐Dahlmann:** conceptualization, writing – original draft, writing – review and editing, methodology, investigation. **Astrid Dempfle:** methodology, writing – review and editing, formal analysis, supervision. **Stefan Eckardt:** data curation, writing – review and editing, methodology. **Josef Neulen:** methodology, writing – review and editing, supervision. **Kelly L. Klump:** conceptualization, supervision, writing – original draft, writing – review and editing.

## Conflicts of Interest

Stefan Eckardt is employed at the Techniker Health Care Service. The other authors declare no conflicts of interest.

## Data Availability

The data that support the findings of this study are available from VdEK. Restrictions apply to the availability of these data, which were used under license for this study. Data are available from the author(s) with the permission of VdEK.
